# PATZ1 is a target of miR-29b that is induced by *Ha-Ras* oncogene in rat thyroid cells

**DOI:** 10.1038/srep25268

**Published:** 2016-04-29

**Authors:** Michela Vitiello, Teresa Valentino, Marta De Menna, Elvira Crescenzi, Paola Francesca, Domenica Rea, Claudio Arra, Alfredo Fusco, Gabriella De Vita, Laura Cerchia, Monica Fedele

**Affiliations:** 1 Institute of Experimental Endocrinology and Oncology (IEOS), National Research Council (CNR), 80131 Naples, Italy; 2 Department of Molecular Medicine and Medical Biotechnologies, University of Naples “Federico II”, 80131 Naples, Italy; 3 National Cancer Institute “Fondazione Giovanni Pascale”, IRCCS, 80131 Naples, Italy

## Abstract

The regulatory transcriptional factor PATZ1 is constantly downregulated in human thyroid cancer where it acts as a tumour suppressor by targeting p53-dependent genes involved in Epithelial-Mesenchymal Transition and cell migration. The aim of the present work was to elucidate the upstream signalling mechanisms regulating PATZ1 expression in thyroid cancer cells. The bioinformatics search for microRNAs able to potentially target PATZ1 led to the identification of several miRNAs. Among them we focused on the miR-29b since it was found upregulated in rat thyroid differentiated cells transformed by the *Ha-Ras* oncogene towards a high proliferating and high migratory phenotype resembling that of anaplastic carcinomas. Functional assays confirmed PATZ1 as a target of miR-29b, and, consistently, an inverse correlation between miR-29b and PATZ1 protein levels was found upon induction of *Ha-Ras* oncogene expression in these cells. Interestingly, restoration of PATZ1 expression in rat thyroid cells stably expressing the *Ha-Ras* oncogene decreased cell proliferation and migration, indicating a key role of PATZ1 in Ras-driven thyroid transformation. Together, these results suggest a novel mechanism regulating PATZ1 expression based on the upregulation of miR-29b expression induced by *Ras* oncogene.

Thyroid cancer is one of the most frequent malignancies of the endocrine system. Moreover, projections of cancer incidence revealed that by 2030 thyroid cancer, together with melanoma and uterine cancer, is going to surpass colorectal cancer and become the second highest diagnosis of cancer in women, and fourth in absolute cases^1^. It includes carcinomas of different degree of differentiation ranging from the papillary thyroid cancer (PTC) and follicular thyroid cancer (FTC), which are well differentiated, through poorly differentiated cancer (PDTC) to anaplastic thyroid cancer (ATC), which is fully undifferentiated and is the most aggressive cancer in the mankind^2^. Thyroid carcinogenesis represents a good multi-step model of cancer disease because the different thyroid cancer histotypes are characterized by distinct arrays of genetic and epigenetic alterations, including somatic mutations, alterations in gene expression patterns, microRNA (miRNA) deregulation and aberrant gene methylation^3^. Most of these alterations activate the Ras signalling cascade. However, Ras mutations are hardly detected in PTC, while they are more frequently found in the follicular variant of PTC, FTC, PDTC and ATC^2^,^4^. We have recently shown that in most of FTC, PDTC and ATC, and less frequently in PTC, the POZ (BTB) and AT hook containing C2H2 zinc finger 1 (PATZ1), is downregulated^5^.

PATZ1, also known as Zfp278, ZSG or MAZ-Related Factor (MAZR), is a regulatory transcription factor able to either activate or repress gene transcription depending on the cellular context^6^,^7^,^8^,^9^. The human PATZ1 gene is located on chromosome 22q12.2 and is transcribed in four alternative spliced variants that give rise to four structurally similar isoforms^7^,^10^. PATZ1 has been reported to play critical roles in spermatogenesis^11^, T cell lineage specification^12^, embryonic development^13^, apoptosis^9^,^14^, proliferation^13^,^15^,^16^, senescence^13^,^17^, stem cell identity^18^, reprogramming^19^, DNA damage response^16^ and cancer, where it seems to have a dual oncogene/tumour suppressor role^5^,^14^,^15^,^16^,^20^. In particular, in thyroid cancer PATZ1 has been shown to act as a tumour suppressor since it is downregulated in a large panel of thyroid cancer samples and cell lines and restoration of its expression in thyroid cancer cells decreased several aspects of the transformed phenotype, including cellular migration, epithelial-mesenchymal transition and *in vivo* tumorigenic potential^5^. Therefore, the aim of our studies has been to unveil the mechanisms that could regulate PATZ1 expression in thyroid cancer. To this purpose, our attention was focused on the miRNAs.

The miRNAs are endogenous single stranded non coding RNAs of about 22 nucleotides in length, which function at post-transcriptional level as negative regulators of gene expression. Several studies have analysed miRNA expression in numerous and different types of thyroid tumours, evidencing a miRNA deregulation in cancer^21^,^22^,^23^,^24^ and the miRNA expression profile presents a significant variability between different kinds of thyroid cancers, even if they originate from the same type of thyroid cells^25^.

Recent studies have demonstrated that in FRTL5, well characterized normal rat thyroid epithelial cells, extensively used to study the molecular mechanisms of neoplastic thyroid transformation *in vitro*, the oncogene *Ha-Ras*^*V12*^ is able to drive cell transformation toward an undifferentiated phenotype, resembling that of ATC and characterized by a high migratory and invasive aptitude^26^,^27^. It has been already shown that the expression of oncogenic Ras in this cell system is able to induce aberrant expression of miRNAs^28^. Among them the top scored upregulated miRNA was miR-21, which has been shown to play an important role in oncogenic Ras-induced cell proliferation *in vitro* and to be regulated in human PTCs^29^,^30^. However, other miRNAs, including miR-29b, appear significantly upregulated downstream of oncogenic Ras^28^. Interestingly, this miRNA is predicted by bioinformatic analysis to be able to target PATZ1. Herein we validated miR-29b targets PATZ1 and found its expression is inversely correlated to PATZ1 protein levels in Ras oncogene transformed thyroid cells. Then, we confirmed the tumour suppressor activity of PATZ1 in thyroid cancer since the restoration of its expression in the Ras-transformed thyroid cells significantly decreased cell proliferation and migration.

## Results

### PATZ1 is a target of miR-29b

In order to identify potentially conserved miRNA being able to down-modulate the expression of PATZ1 protein, we used the www.microRNA.org web platform that is based on the miRanda application^31^ and uses the mirSVR predicted target site scoring method, giving a down-regulation score and identifying a significant number of experimentally determined non-canonical and non-conserved sites^32^. The human *PATZ1* gene expresses 4 major alternatively spliced transcripts: PATZ1-001, -004 and -002, sharing a common 3′-UTR (NM_014323.2/NM_032050.1) that in PATZ1-002 includes 372 additional nucleotides (NM_032052.1), and PATZ1-003, which has a completely different 3′-UTR (NM_032051.1) (see Supplementary Fig. S1).

**Figure 1:**
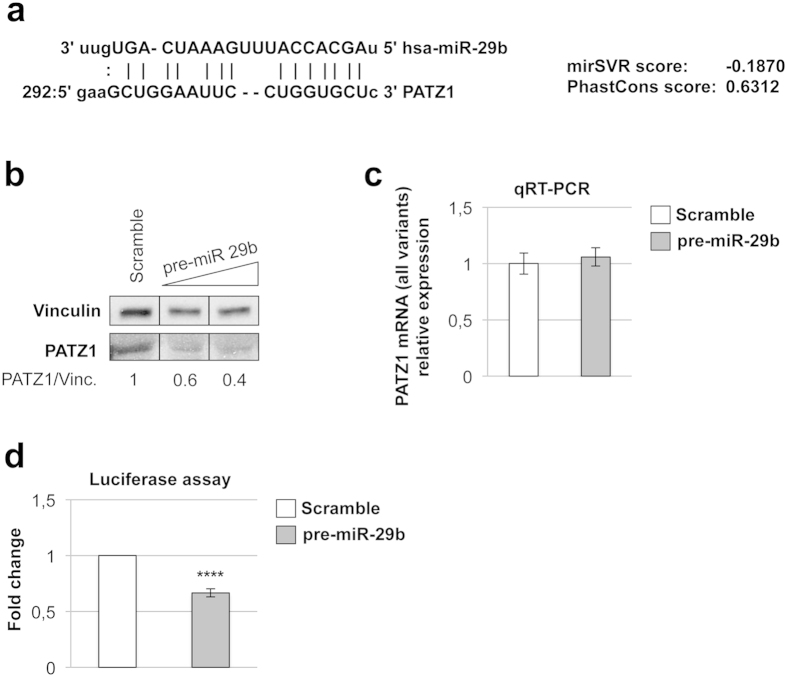
Validation of PATZ1 as a target of miR-29b. (**a**) predicted miR-29b/ PATZ1 alignment, according to microRNA.org web system. mirSVR (cutoff 0.1 or lower) and PhastCons (cutoff 0.57 or higher) are downregulation and conservation scores, respectively. (**b**) Western blot using anti-PATZ1 on HEK293 total cellular extracts collected 72 h after transfection with increasing amount (50–100 nM) of synthetic miR-29b precursor or scramble (100 nM) oligonucleotide. Vinculin was used for normalization. Relative expression levels, compared to scramble-transfected control and normalized with respect to vinculin, are indicated on the bottom. Black lines delineate the boundary between not contiguous lanes of the same gel. (**c**) qRT-PCR on total RNA from HEK293 cells previously transfected with 100 nM synthetic miR-29b precursor or scramble oligonucleotide. PATZ1 mRNA levels were normalized for endogenous G6PD levels. The mean ± SE of four independent experiments performed in duplicate is reported. (**d**) Luciferase assay on HEK293 cells co-transfected with the Luc-PATZ1-3′UTR and pCMV renilla reporter vectors along with 100 nM synthetic miR-29b precursor or scramble oligonucleotide. Relative firefly luciferase activity levels were normalized for renilla luciferase activity and analysed relatively to scramble-transfected cells, which were set to 1. The mean ± SE of four independent experiments performed in duplicate is reported. ****P < 0,0001.

This analysis identified several miRNAs as potential PATZ1-targeting miRNAs (see Supplementary Fig. S1 for the entire list of predicted miRNAs). As indicated by the PhastCons score higher than cutoff, the targeting sites of these PATZ1-targeting miRNAs on the 3′-UTR of PATZ1 are extremely conserved among different species. Here, we focused on miR-29b since it has been previously found up-regulated in thyroid cell proliferation^33^ and transformation^28^. In Fig. 1a, we show the miR/PATZ1 alignment: the mirSVR downregulation score is −0.1870, predicting PATZ1 as a very likely candidate target of miR-29b. Then, to validate such prediction we transfected HEK293 cells with miR-29b synthetic precursor and evaluated PATZ1 protein levels by western blotting after 72 hours. The different alternative splice variants of PATZ1 are differently expressed in HEK293 cells (see Supplementary Fig. S2) and only the most abundant isoform (PATZ1-004) appears detectable by western blot (Fig. 1b). PATZ1 protein levels were reduced up to the 60% in cells transfected with miR-29b in comparison with the cells transfected with the scrambled oligonucleotide. Conversely, quantitative real-time reverse transcription PCR (qRT-PCR) experiments, using primers that amplify a region common to all variants, did not show any changes in PATZ1 mRNA levels indicating that miR-29b does not act on mRNA degradation (Fig. 1c). Next, to further assess the inhibition of PATZ1 protein by miR-29b, we used a PGL-3-CTRL vector containing the 3′-UTR sequence common to the hPATZ1-001 and 004 transcripts, which includes a predicted miR-29b target site, cloned downstream the firefly luciferase gene. This reporter vector was transfected in HEK293 cells along with synthetic precursor of miR-29b, or scramble miRNA control, and luciferase activity was assessed 72 h after the transfection. As shown in Fig. 1d, overexpression of miR-29b significantly reduced luciferase activity of the reporter vector, compared to scramble transfected controls, suggesting that the inhibition of PATZ1 protein expression by miR-29b acts, either in a direct or an indirect manner, on the 3′-UTR of PATZ1. These data are consistent with data extracted from the DIANA-TarBase v7.0 database^34^, where PATZ1 is indicated as an experimental validated target of has-miR-29b-3p as assessed by immunoprecipitation in BCBL-1 lymphoma cells^35^.

**Figure 2:**
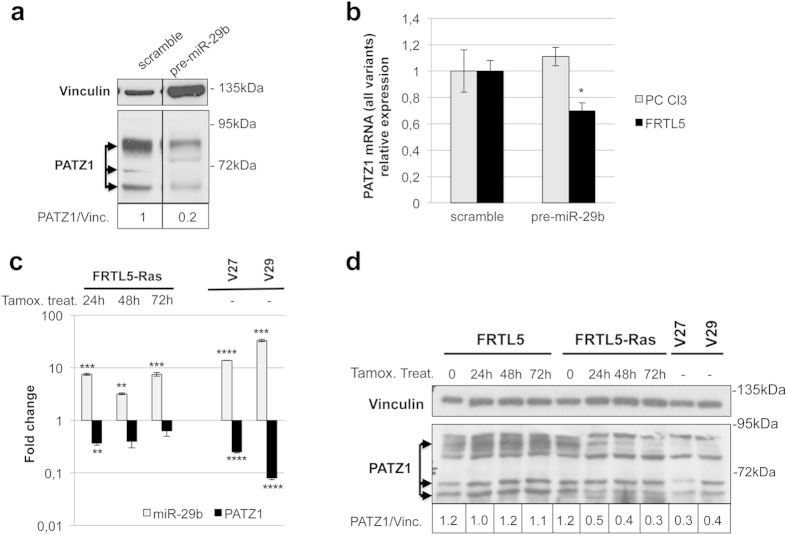
miR-29b targeting of PATZ1 in rat thyroid cells. (**a**) Western blot using anti-PATZ1 on PC Cl3 total extracts previously transfected with synthetic miR-29b precursor or scramble oligonucleotide. Three major specific bands were observed (arrows). Vinculin was used for normalization. Densitometric analysis by Image J software was applied on the gel: Relative expression levels of PATZ1, compared to scramble-transfected control and normalized with respect to vinculin, are indicated on the bottom. Black lines delineate the boundary between not contiguous lanes of the same gel. (**b**) qRT-PCR on total RNA from PC Cl3 and FRTL-5 cells previously transfected with synthetic miR-29b precursor or scramble oligonucleotide. PATZ1 mRNA levels were normalized for endogenous G6PD levels. The mean ± SE of three independent experiments performed in duplicate for each cell line is reported. *P < 0.05 compared with scramble transfected control. (**c**) qRT-PCR on total RNA from FRTL-5-Ras inducible cells treated with Tamoxifen at the indicated times and FRTL-5 clones V27 and V29 stably expressing the Ha-Ras V12 oncogene. miR-29b and PATZ1 expression levels were normalized for endogenous U6 and G6PD levels, respectively. The mean ± SE of one experiment performed in triplicate and three independent experiment performed in duplicate is reported for miR-29b and PATZ1, respectively. **P < 0.01; ***P < 0.001; ****P < 0.0001 compared with mock-treated or mock-transfected cells for FRTL5-Ras inducible cells and stable clones, respectively. (**d**) Western blot using anti-PATZ1 on total extracts from cell as in C. The three specific bands corresponding to different PATZ1 isoforms are indicated by arrows. Normalized expression levels of PATZ1, as assessed by densitometric analysis on the upper PATZ1-specific band with respect to vinculin expression, are indicated on the bottom.

The ability of miR-29b to target *PATZ1* was also confirmed in a thyroid cell system using the rat thyroid cell line PC Cl3. Indeed, PATZ1 protein levels were downregulated following thyroid cell transfection with miR-29b synthetic precursor (Fig. 2a). We observed three specific signals likely corresponding to different isoforms and/or post-translational modification of the rat PATZ1 protein (see Supplementary Fig. S2 for protein homology between human and rat isoforms and relative expression of each mRNA variant). All these signals were drastically reduced (80% reduction) in thyroid cells overexpressing miR-29b, suggesting that all expressed PATZ1 variants may be targets of this miRNA. Interestingly, differently from HEK293 and PC Cl3 cells, transfection of miR-29b precursor into FRTL5 cells, another rat thyroid cell line, resulted in downregulation of PATZ1 at mRNA level (Fig. 2b). In FRTL5 cells, only the two transcript variants rPatz1-203 and -204 appear significantly expressed (see Supplementary Fig. S2). Both these variants were downregulated after transfection of the miR-29b (see Supplementary Fig. S3).

### miR-29b and PATZ1 expression levels are inversely correlated in thyroid cells expressing the Ha-Ras^V12^ oncogene

Interestingly, miR-29b is one of the most upregulated miRNAs in FRTL5 following expression of a Ras oncogene in an inducible cell system, in which a chimeric form of Ha-Ras oncoprotein, ER-Ras, can be activated by tamoxifen^26^, as assessed by screening the www.microRNA.org web platform (see Supplementary Fig. S4). Notably, it was the miR-29 isoform more expressed in this Ras-driven rat thyroid cell transformation system. To explore a possible interaction between miR-29b and PATZ1 downstream of oncogenic Ras, we analysed the expression of PATZ1 and miR-29b in the same tamoxifen-inducible FRTL5 cell system, in which the expression of the *Ha-Ras*^*V12*^ oncogene was induced at different time points. As shown in Fig. 2, consistent with the data extracted from the above website, miR-29b was upregulated following induction of Ha-Ras^V12^ as early as after 24 h of treatment with tamoxifen (Fig. 2c). Conversely, PATZ1 was down-regulated after treatment with tamoxifen at both mRNA (Fig. 2c) and protein levels (Fig. 2d), confirming the functional miR-29b/PATZ1 interaction in thyroid cells. To rule out the possibility that such effects could be due to tamoxifen treatment, we treated both ER-Ras FRTL5 and parental FRTL5 cells with tamoxifen, showing that miR-29b is induced and PATZ1 downregulated only in the presence of oncogenic Ras activity (Fig. 2d). The expression of miR-29b and PATZ1 was also analysed in two previously established independent clones of FRTL5 cells stably expressing high levels of the human *Ha-Ras*^*V12*^ oncogene^26^. Also in these cell clones miR-29b was upregulated and PATZ1 downregulated, showing that miR-29b overexpression and PATZ1 downregulation are persistent events in chronic thyroid cell transformation induced by Ras oncogene. Interestingly, the mRNA levels of PATZ1 in Ha-Ras^V12^ stable clones show a perfect negative correlation (r = −1) with the levels of miR-29b (Fig. 2c). As these cells are transformed by the native human *Ha-Ras* oncogene, it is ruled out the possibility that induction of miR-29b, as well as downregulation of PATZ1 could be an artefact of the chimeric Ras oncoprotein. Taking together the qRT-PCR results from both the inducible and stable clones we found a strong negative correlation (r = −0.797), meaning a functional dependence between miR-29b and PATZ1 expression. All together these results confirm that miR-29b targets PATZ1 in thyroid cells and suggest that PATZ1 is a downstream effector of the oncogenic Ras signalling.

### PATZ1 expression decreases proliferation and migration of Ras-transformed thyroid cells

**Figure 3:**
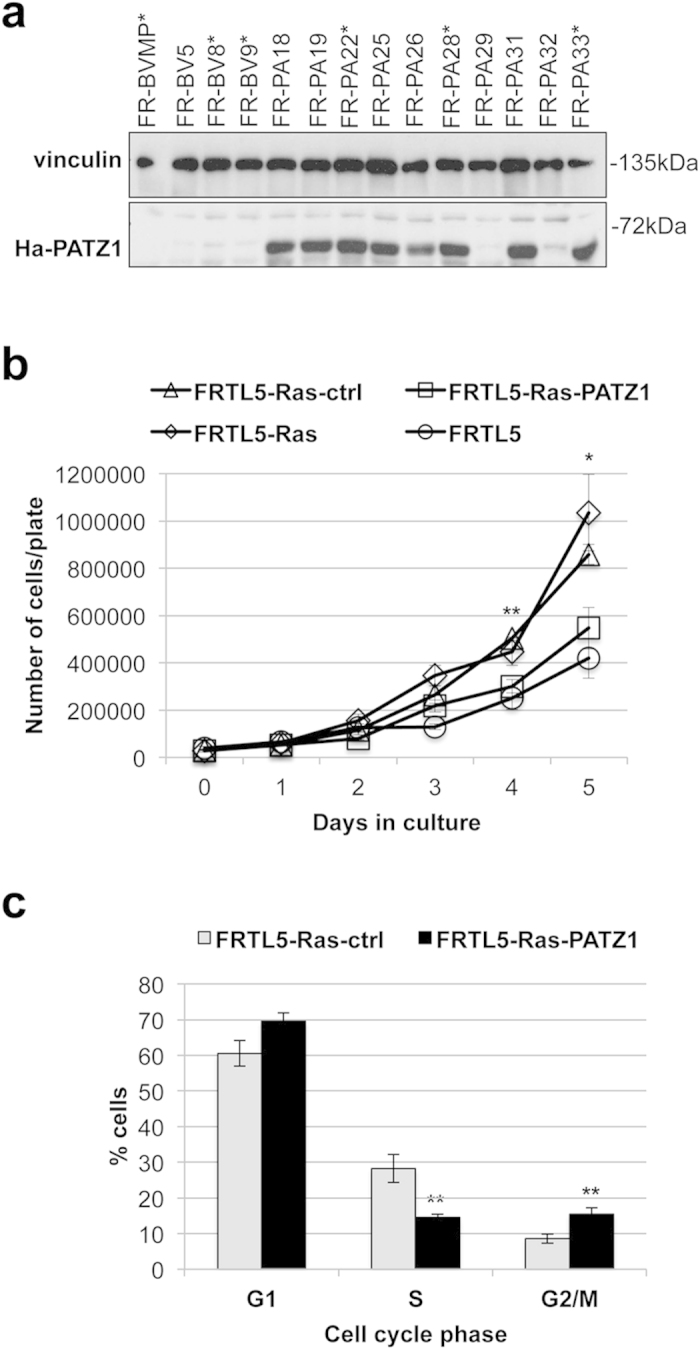
Inhibition of proliferation in PATZ1-transfected FRTL5-Ras cells. (**a**) Western blot analysis of PATZ1 in selected clones and mass populations (MP) of FRTL5-Ras-V29 cells transfected with PATZ1 (FR-PA) or the backbone vector (FR-BV). Asterisks indicate the mass populations and/or clones selected for the following experiments. (**b**) Growth curves on different stably expressing PATZ1 cell clones and/or mass populations of FRTL5-Ras cells (FRTL5-Ras-PATZ1) compared to controls expressing the empty vector (FRTL5-Ras-ctrl). Mean values ± SE of three clones or mass populations for each cell line are reported. FRTL5 and parental FRTL5-Ras-V29 (FRTL5-Ras) cells were also analysed for comparison of the results. (**c**) Flow cytometry (FACS) analysis on proliferating cells. Mean values ± SE of at least 3 independent experiments performed in two independent cell clones for each cell line are reported as percentages of the cell cycle distribution. *P < 0.05; **P < 0.01.

To investigate whether the downregulation of PATZ1 plays a role in Ras-induced thyroid cell transformation, we restored PATZ1 expression in Ras-transformed rat thyroid cells by transfecting a PATZ1-expressing construct in FRTL5 cells stably expressing the oncogenic Ha-Ras^V12^ (Fig. 3a). Three cell clones efficiently expressing transfected PATZ1 (FR-PA22, FR-PA28, FR-PA33) and three controls transfected with the backbone vector (FR-BVMP, FR-BV8, FR-BV9) were selected and functionally studied. We, first, analysed cell growth rate by performing growth curves. As reported in Fig. 3b, FRTL5-Ras-PATZ1 cells showed a significant decrease in the proliferation rate compared to control cells (FRTL-Ras-ctrl). Interestingly, the growth rate of FRTL5-Ras-PATZ1 was not different from that of untransformed FRTL5 cells. On the other side, as expected, FRTL5-Ras-ctrl growth curves were undistinguishable from those of parental cells (FRTL5-Ras). Cell cycle analysis performed on proliferating cells confirmed such hypothesis, showing a significant decrease in S phase and increase in G2/M phase of PATZ1-expressing clones compared to Ras-transformed backbone transfected cells (Fig. 3c). Therefore, these results indicate that PATZ1 represents a new downstream negative effector of Ras-induced enhancement of cell proliferation in thyroid transformed cells.

**Figure 4:**
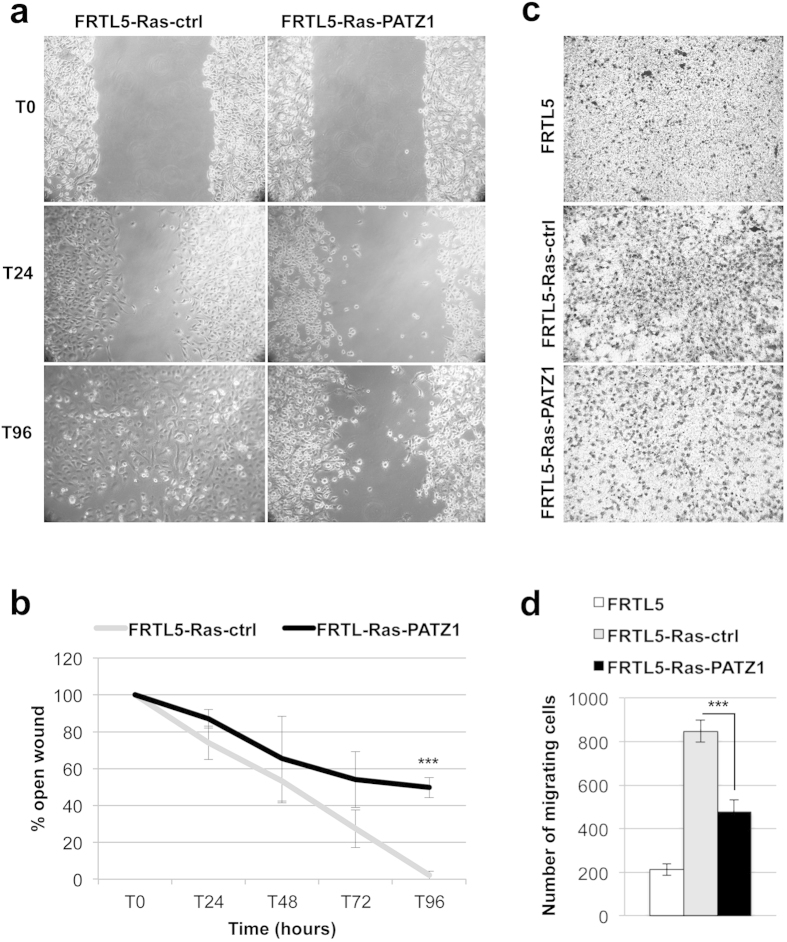
Inhibition of migration in PATZ1-transfected FRTL5-Ras cells. (**a**) Representative images of a wound healing assay in control (FRTL-Ras-ctrl) and PATZ1-expressing FRTL5-Ras cells at 0, 24 h and 96 h after a confluent cell monolayer was wounded. (**b**) Percent of open wound, calculated as mean values ± SE of three independent cell clones or mass populations for each cell line, at 0 h, 24 h, 48 h, 72 h and 96 h after wound scratch. ***P < 0.001. (**c**) Representative images of a Transwell assay performed on FRTL5, FRTL5-Ras-ctrl and FRTL5-Ras-PATZ1 cells. Dark cells (stained with crystal violet) represent migrating cells. (**d**) Number of migrating cells expressed as mean values ± SE of three clones for each transfected construct. ***P < 0.001.

It has been reported that the Ras oncogene is also able to induce cell migration^27^,^36^,^37^ that accounts for its key role in thyroid cancer progression^38^. Therefore, to investigate whether PATZ1 re-expression affects the migratory capability of FRTL5-Ras cells, we performed wound-healing and Transwell migration assays. Specifically, a confluent cell monolayer was scratched and the wound was photographed at different time points (Fig. 4a,b). For all time points cells were serum starved, thus avoiding the possibility that the effect on cell proliferation could interfere with the read out of the assay. As shown in Fig. 4a,b, the migration capacity of PATZ1-transfectants was significantly reduced compared with that of control cells. However, the wound-healing assay is particularly suitable for studying the effects of cell-matrix and cell-cell interactions on cell migration, but does not give insights on migration in response to a particular chemical signal, which is usually referred to as chemotaxis. To better investigate this issue we performed a Transwell assay, analysing cell migration across 8-μm membrane pores (Boyden chambers) in response to Bovine Serum (BS) plus 6H. The results of this further migration assay were in agreement with those of the wound-healing assay, showing for all the three selected PATZ1-expressing clones a strong and statistically significant reduction in their migration capability of PATZ1-expressing clones compared to controls (Fig. 4c,d).

All together these functional assays confirm that restoration of PATZ1 expression is able to decrease both proliferation and migration of thyroid transformed cells as already described in human thyroid cancer cells^5^.

## Discussion

Our research group has recently reported that PATZ1 is downregulated in thyroid cancer tissues compared to normal thyroid and exerts a tumour-suppressor role mainly through the regulation of p53-target genes *EpCam*, *RhoE* and *Caldesmon*, thus resulting in reduced migration and invasion *in vitro*, as well as mesenchymal-epithelial transition and reduced tumour growth *in vivo*^5^. Then, we questioned for the molecular mechanism underlying the downregulation of PATZ1 in thyroid carcinogenesis focusing on miRNAs. Indeed, they represent nowadays the actual challenge in both diagnostic and therapy for biomedical purposes, and their deregulation in thyroid cancer has been widely demonstrated^21^,^22^,^23^,^24^,^25^. By bioinformatic analysis we identified different miRNAs predicted to target PATZ1. Among them, we concentrated on miR-29b since it was one of the most upregulated miRNAs in FRTL5 rat thyroid cells following expression of the *Ha-Ras*^*V12*^ oncogene^28^. Moreover, it has also been reported that miR-29b expression is upregulated in rat thyroid cells following cell growth induced by thyreotropin and its overexpression promotes thyroid cell proliferation. Consistently, increased miR-29b expression was found in experimental murine PTU-induced and human goiters, thus indicating its upregulation as a critical event in the regulation of thyroid cell proliferation^33^.

Here, we show that enforced miR-29b expression in thyroid and non-thyroid cells significantly reduces PATZ1 protein levels. Conversely, PATZ1 transcript levels were significantly downregulated only in FRTL5 cells, suggesting that miR-29b ability to reduce PATZ1 mRNA stability is cell context-dependent. Then, luciferase assays using the 3′UTR of PATZ1 demonstrated that this effect is specifically mediated by this regulatory region of the PATZ1 gene. In agreement with the ability of miR-29b to target PATZ1, we found that PATZ1 and miR-29b expressions were inversely correlated at both RNA and protein level in a thyroid cell system in which the expression of the oncogenic Ha-Ras^V12^ was induced by a tamoxifen-inducible construct, as well as in FRTL5 cells in which Ha-Ras^V12^ was stably expressed. From these data we can assume that miR-29b has oncogenic activity by downregulating the tumour suppressor activity of PATZ1, in agreement with the ability of miR-29b to enhance cell migration and invasion in nasopharyngeal carcinoma progression by regulating SPARC and COL3A1 expression^39^. Moreover, miR-29b enhances migration of human breast cancer cells and is overexpressed in breast metastases in comparison with the breast cancer tissue^40^. Still, miR-29b is expressed at higher levels in indolent human B-cell CLL (chronic lymphocytic leukaemia) with respect to normal CD19 + B cells and, consistently, transgenic mice overexpressing miR-29b in B cells developed B-CLL^41^. However, it is worth to note that miR-29b acts as a tumour suppressor in other cancer tissues. Indeed, mir-29b inhibits the progression of esophageal squamous cell carcinoma by targeting MMP-2^42^. In breast cancer, miR-29b expression is negatively associated with the HER2 sub-type, and its overexpression inhibits breast cancer cell proliferation and induces apoptosis mainly downregulating STAT3 protein levels^43^. Equally, miR-29b expression inhibits glioblastoma cell proliferation, migration, invasion, angiogenesis and stemness maintenance, while promoting apoptosis, by targeting DNMT3A-3B and BCL2L2^44^,^45^. Similar tumour suppressor activity for miR-29b in colon^46^, lung^47^ and ovarian^48^ cancer tissues was reported. It is likely that the cellular context determines the oncogenic or the antioncogenic activity of miR-29b, as previously reported for other miRNAs^49^. Interestingly, PATZ1 role in cancer also appears to be cell context-dependent, and in glioblastoma and colon cancer cells, where miR-29b acts as tumour suppressor^44^,^45^,^46^, PATZ1 behaves as an oncogene^14^,^15^.

Since, we did not find upregulation of miR-29b expression in human thyroid samples in which PATZ1 was downregulated (data not shown) it is possible that, similarly to what occurs for other tumour suppressor genes silenced by oncogenic RAS^50^,^51^, the Ras-directed downregulation of PATZ1 proceeds in two steps: PATZ1 may be first downregulated by miR-29b in a Ras-driven reversible step. Then, as the transformation progresses, PATZ1 becomes permanently downregulated by means of other epigenetic events through a specific complex pathway initiated by Ras oncogene. However, Independently from the role of miR-29b, the results reported here suggest that PATZ1 may be a negative effector of the oncogenic Ras signalling in thyroid carcinogenesis. In this frame, it is worth to note that, similarly to PATZ1 that is further downregulated in FTCs, PDTCs and ATCs compared to PTCs^5^, Ras mutations are more frequently detected in FTCs, PDTCs and ATCs than in PTCs^2^. In order to deepen a causal role of PATZ1 down-regulation in thyroid carcinogenesis induced by oncogenic Ras, we overexpressed PATZ1 in Ras-transformed FRTL5 cells, finding a significant impairment of different cellular functions related to the transformed phenotype, including proliferation and migration. Therefore, it appears that PATZ1 downregulation is indeed a crucial step in Ras oncogenic signalling, acting in different processes of cellular transformation induced by activated Ras. These data are also consistent with the role of PATZ1 to regulate the same cellular processes in human thyroid cancer cell lines^5^. Notably, given the new role recently unveiled for PATZ1 in DNA damage response^16^, it could be hypothesized that the abrogation of the DNA damage response caused by acute expression of activated Ras in normal rat thyroid cells^52^, occurs through downregulation of PATZ1 expression.

Overall, our work demonstrates that PATZ1 is a target of miR-29b and a pivotal regulator acting downstream of oncogenic Ras to suppress thyroid cell transformation.

## Methods

### Bioinformatic analysis

For the identification of miRNAs potentially targeting PATZ1, the miRanda-mirSVR algorithm (available at www.microrna.org) has been used. It is based on a combination of specific-base pairing rules and conservational analysis to score possible match between 3′UTR of specific genes with several miRNAs, using a dynamic programming algorithm weighted to favour 5′ complementarity to enumerate initial target sites. The method uses two scores: mirSVR (cutoff of −0.1 or lower), which predicts likelihood of target mRNA down-regulation; and Phast Cons (cutoff of 0,57 or higher), which indicates the probability that each nucleotide belongs to a conserved element^32^.

### Cell culture, Transfections and plasmids

Continuous rat thyroid cell lines FRTL5, FRTL5-Ras and PC CL3 were cultured in Ham’s F-12 medium and Coon’s modification supplemented with 5% CS, 1% L-glutamine, 1% penicillin/streptomycin (GIBCO-BRL) and in the presence of a mix containing six growth factors, 6H (10 nM TSH, 10 nM hydrocortisone, 100 nM insulin, 5 mg/ml transferrin, 5 nM somatostatin, and 20 μg/ml glycyl-histidyl-lysine), in a 5% CO2 atmosphere. Human embryonic kidney HEK293 cells were cultured in DMEM supplemented with 10% FBS, 1% L-glutamine and 1% penicillin/streptomycin (GIBCO-BRL) in a 5% CO2 atmosphere. The selected cell clones of FRTL5-Ras cells and the control cells were cultured in Ham’s F-12 medium and Coon’s modification supplemented with 5% CS, 1% L-glutamine, 1% penicillin/streptomycin (GIBCO-BRL) and in the presence of a mix containing 6H, in a 5% CO2 atmosphere.

HEK293 transfections were performed by Lipofectamine 2000 (Invitrogen) according to manufacturer’s instruction, with 100 nM Scramble or 100 nM miR-29b miRNA precursors (Ambion, Austin, TX) together with PGL-3-CTRL vector or the PATZ1-3′UTR luciferase reporter plasmid. For the PATZ1-3′UTR luciferase reporter construct, the 1098 bp 3′UTR region of the h*PATZ1*-001/004 transcripts, was amplified from HEK293 cells DNA by using the following primers: Fw PATZ1-001/004 3′UTR: 5′-ATATGATATCGGCAGCTGCTGTGTCC-3′ and Rev PATZ1 3′UTR V1/2 5′-GCATGATATCGTACAAACATTTTTAAT-3′.The amplified fragment was cut with EcoRV and cloned into pGL3-Control firefly luciferase reporter vector (Promega, Madison, Wisconsin, USA) at the XbaI site. For stable transfection FRTL5-Ras cells were transfected by Fugene6 (Roche) according to manufacturer’s instruction with HA-PATZ1 plasmid encoding for PATZ1 variant 4 or the empty vector pCEFL-HA, both expressing the gene for the resistance to neomycin. Stable transfectants were clonally selected in medium with 1 μg/ml neomycin (G418) (Life Technologies) for 10 days, and cell clones were screened for PATZ1 expression by qRT-PCR and Western blot analysis.

### Protein extraction, Western blotting and antibodies

Cells were lysed in buffer containing 1% Nonidet P-40, 1 mmol/liter EDTA, 50 mmol/liter Tris-HCl (pH 7.5), and 150 mmol/liter NaCl supplemented with Complete protease inhibitors (Roche Applied Science). Total proteins were resolved in a 8% polyacrylamide gel under denaturing conditions and transferred to nitrocellulose filters for Western blot analyses. Membranes were blocked with 5% BSA in TBS and incubated with the primary antibodies. Membranes were then incubated with the horseradish peroxidase–conjugated secondary antibody (1:3.000) and the reaction was detected with a Western blotting detection system (enhanced chemiluminescence; GE Healthcare). The primary antibodies used are anti-PATZ1 antibody (polyclonal antibody raised against a conserved peptide recognizing all PATZ1 isoforms of rat, mouse and human origin). To ascertain that equal amounts of protein were loaded, the membranes were incubated with antibodies against the anti-vinculin protein (sc-7649) (Santa Cruz Biotechnology, Santa Cruz, CA).

### RNA extraction and qRT-PCR analysis

Total RNA was extracted using TRI-reagent solution (Sigma, St Louis, MO, USA) and treated with DNase (Invitrogen). Reverse transcription was performed according to standard procedures (Qiagen, Valencia, CA). qRT-PCR analysis was performed using the Power SYBR Green PCR Master Mix (BioRad) according to manufacturers’ instructions with the following primer sequences to amplify the indicated genes: human *PATZ1* all variants (5′-TACATCTGCCAGAGCTGTGG-3′/5′-TGCA CCTGCTTGATATGTCC-3′); hPATZ1-001 (5′- CAGGTCTCCAGGCACCAG -3′/5′- TGAGCATTTCTGGC CTTCTT -3′); hPATZ1-004 (5′- CATACATGGCAGACCACCTG -3′/5′- AATCGGATCCTGATGTGAGC -3′); hPATZ1-002 (5′- CATACATGGCAGACCACCTG -3′/5′- GCCTGGAGACCTCGGTTAC -3′); hPATZ1-003 (5′- CTGAGCGGCCTCACAAGT -3′/5′- GTCGCTAGGAAGAGGTTCCA -3′); human *G6PD* (5′-GATCTACCGCATCGACCACT -3′/5′-AGATCCTGTTGGCAAATCTCA -3′); rat *G6PD* (5′- TCCTCTA TGTGGAGAATGAACG -3′/5′- TCATTCAGAGCTTTGCCACA -3′); rat *PATZ1* all variants (5′- CCAGAGCTGTGGGAAAGG -3′/5′- TGCACCTGCTTGATATGTCC -3′); rPatz1-202 (5′- CTGGAACCTCTTCCTAGCGA -3′/5′- TGTGCTTCTTCAGGTGGTCA -3′); rPatz1-203 (5′- GAGGGGCCCAGCAACTTC -3′/5′- ATAGGTCCTGGCGCAGTG -3′); rPatz1-204 (5′- CTGAGCGCCCTCACAAATG -3′/5′- GCGGTATCAGGAACTGAA -3′). Primers to amplify miR-29b and U6 snRNA were purchased from Qiagen. To calculate the relative expression levels we used the 2-ΔΔCT method^53^. Glucose-6-phosphate dehydrogenase (G6PD) and U6 were used as normalizers for genes and miRNAs, respectively.

### Luciferase assay

For Luciferase assays, the pCMV-Renilla plasmid (Promega, Mannheim, Germany) was co-transfected with 3′UTR region of PATZ1 Variant 1/2. Luciferase and Renilla activities were assessed with the Dual-Light Luciferase system (Promega), according to the manufacturer’s protocol, 72 h after the transfection. Luciferase activity was normalized for the Renilla activity.

### Proliferation and Migration assays

For the growth curves the cells (3 × 10^4^ cells/dish) were plated in a series of 60-mm culture dishes and counted daily for 5 consecutive days through the Bürker chamber. The count was performed in the presence of Trypan blue, a dye that penetrate in cells that have lost membrane integrity and which shows, therefore, dying cells.

To detect the changed capacity of tumour cell migration, we performed a wound-healing assay. Specifically, cells were digested with 0.25% trypsin and adjusted for a concentration of 5 × 10^5^ cells/ml of cell suspension, and then inoculated into 60 mm plates and cultured at 37 °C overnight. In the next day, cells were cultured in serum-free medium, reached approximately 95–100% confluence, and cell monolayer was wounded by 20 μl tips. The cells were incubated for 96 h. At 0 h, 24 h, 48 h, 72 h, and 96 h, cells were photographed under an inverted microscope. The migration assay was conducted using plates Transwell cell culture chambers according to described procedures (Corning Costar Corp., Cambridge, MA). Briefly, confluent cell monolayers were harvested with trypsin/EDTA, centrifuged at 1.200 rpm for 5 min, resuspended in medium without serum and without 6H and plated (5 × 10^4^ cells) to the upper chamber of a polycarbonate membrane filter of 8 μM pore size. The lower chamber was filled with complete medium. The cells were then incubated at 37 °C in a humidified incubator in 5% CO2 for 24 h. Non migrating cells on the upper side of the filter were wiped off and migrating cells on the reverse side of the filter were stained with 0.1% crystal violet in 20% methanol for 30 min, washed in PBS 7.4 (137 mM NaCl; 2.7 mM KCl, 4.3 mM NaH2PO4), photographed and counted.

### Flow cytometry analysis

For flow cytometric analysis 2 × 10^5^ cells were plated and analysed after 24 h under normal culture conditions. Briefly, cells were washed once with PBS, and fixed for 2 h in cold ethanol (70%). Fixed cells were washed once in PBS and stained with 50 μg/ml propidium iodide (Roche, Indianapolis, IN) in presence of 40 μg/ml ribonuclease A. Stained cells were analyzed with a flow cytometer (Accuri™ C6 flow cytometer, BD Biosciences, East Rutherford, New Jersey). The data were analysed using a BD Accuri C6 software.

### Statistical analysis

All the experiments were performed in independent biological triplicates or quadruplicates and the results are expressed as the mean ± standard error (SE). Correlation was analysed by Pearson’s correlation test. Means of two groups of data were compared by Student’s t-test. P-value < 0.05 was considered to be statistically significant.

## Additional Information

**How to cite this article**: Vitiello, M. *et al*. PATZ1 is a target of miR-29b that is induced by *Ha-Ras* oncogene in rat thyroid cells. *Sci. Rep.* **6**, 25268; doi: 10.1038/srep25268 (2016).

## Supplementary Material

Supplementary Information
