# Supplementary material for: PATZ1 is a target of miR-29b that is induced by Ha-Ras oncogene in rat thyroid cells
Source: Sci Rep. 2016 Apr 29;6:25268. doi: 10.1038/srep25268 (PMC4850481; doi:10.1038/srep25268)
Supplement: Supplementary Information [file srep25268-s1.pdf]

PATZ1 is a target of miR-29b that is induced by *Ha-Ras* oncogene in rat thyroid cells

List of investigators: Michela Vitiello, Teresa Valentino, Marta De Menna, Elvira Crescenzi, Paola Francesca, Domenica Rea, Claudio Arra, Alfredo Fusco, Gabriella De Vita, Laura Cerchia & Monica Fedele

Table of contents:

Supplementary Figures:

Supplementary Figure S1. Schematic representation of the PATZ1 3'UTRs of the human PATZ1 transcript variants and list of miRNAs predicted to target PATZ1

Supplementary Figure S2. PATZ1 variants expression and homology in human and rat cells.

Supplementary Figure S3. miR29b effect on PATZ1 variants expressed in FRTL5 cells.

Supplementary Figure S4. miR-29 isoforms expression in FRTL5 cells and FRTL5-Ras cells.

A

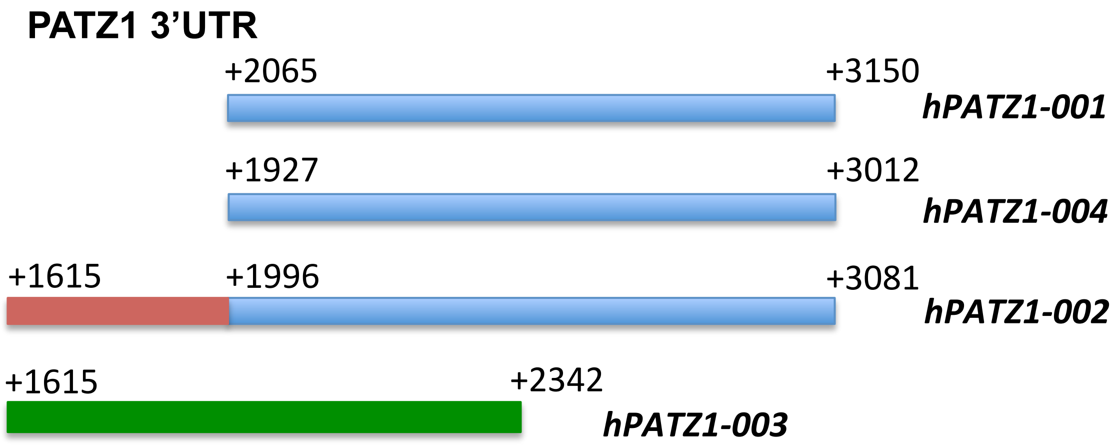

B

hPATZ1-001/-004	hPATZ1-002	hPATZ1-003
miR-22, miR-491-5p, miR-216b, miR-361-5p, miR-544, miR-339-5p, miR-142-3p, miR-29a/b/c, miR-376a/b, miR-24, miR-421, miR-653, miR-134, miR-200b/c, miR-429, miR-495, miR-590-3p, miR-448, miR-153, miR-490-3p, miR-23a/b, miR-383, miR-543	miR-1271, miR-96, miR-136, miR-26a/b, miR-1297, miR-185, miR-22, miR-491-5p, miR-216b, miR-361-5p, miR-544, miR-339-5p, miR-142-3p, miR-29a/b/c, miR-376a/b, miR-24, miR-421, miR-653, miR-134, miR-200b/c, miR-429, miR-495, miR-590-3p, miR-448, miR-153, miR-490-3p, miR-23a/b, miR-383, miR-543	miR-365, miR-150, miR-18a/b, miR-31, miR-138, miR-491-5p. miR-107, miR-103, miR-340, miR-590-3p

Supplementary Figure S1. (A) schematic representation of the PATZ1 3'UTRs of the four human *PATZ1* transcript variants, annotated according to ENSEMBL. Blue bars indicate a common region present in hPATZ1-001, -004 and -002 isoforms. Red and green bars indicate unique sequences present in hPATZ1-002 and -003 isoforms, respectively. The location of the 3'UTR regions with respect to the start coding region of the gene are indicated on each bar (B) miRNAs predicted to target PATZ1, as assessed by the www.microRNA.org websyte.

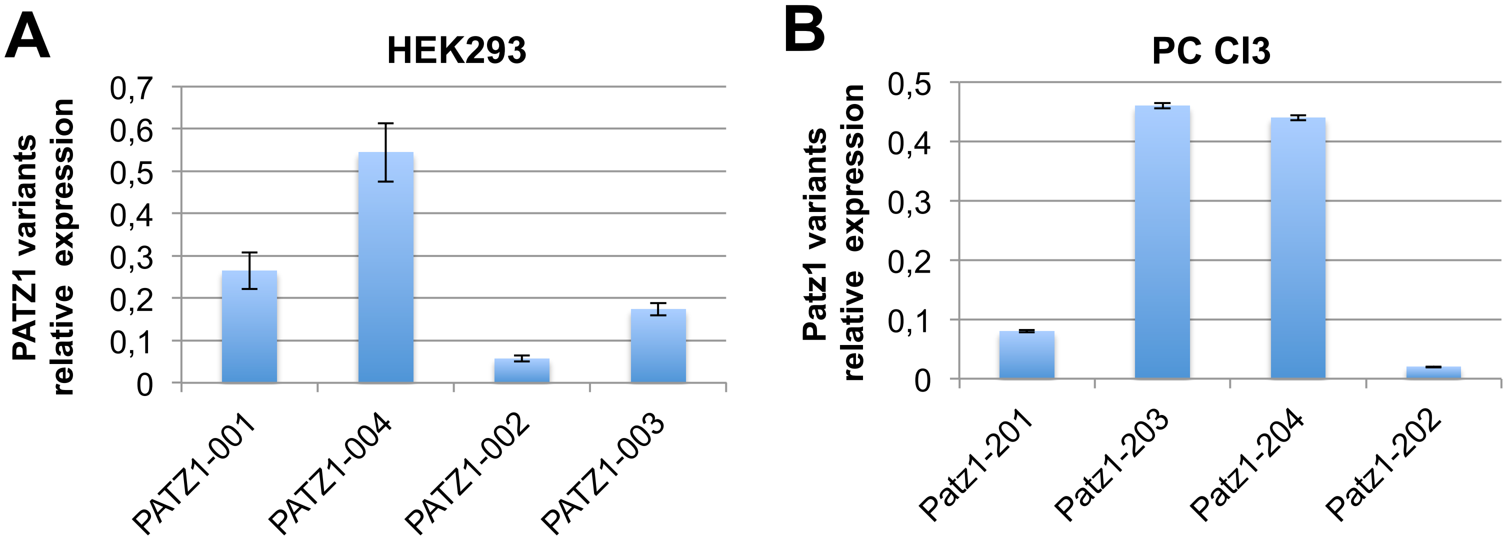

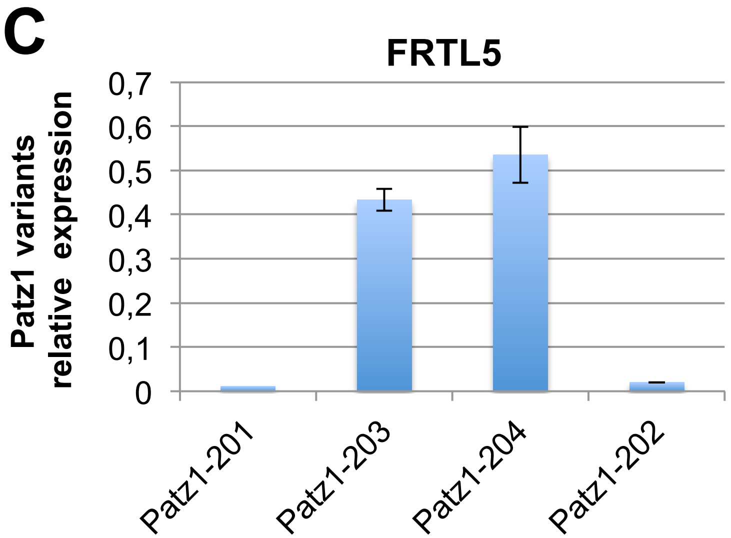

D

hPATZ1	rPatz1	Protein homology	3’UTR homology
-001[a]	-201[a]	92%	62%
-004[a]	-201[a]	99%	62%
-002[a]	-203[a]	99%	69%
-003	-204	99%	n.s.s.
-003	-202	92%	n.s.s.

Supplementary Figure S2. PATZ1 variants expression and homology in human and rat cells. (A-C) qRT-PCRs using exon-specific primers able to discriminate the different variants in HEK293 (A), PC Cl3 (B) and FRTl5 (C) cells. The reported results (mean values + SD of at least three experiments) are relative to amplification of all variants that was set to 1. Only for Patz1-201 variant the results are extrapolated by the difference between expression of all variants and the sum of the other three variants. (D) Protein and 3’UTR homology between human (hPATZ1) and rat (rPatz1) PATZ1 isoforms. [a] = variant with a predicted miR-29b target site in its 3’UTR. n.s.s. = no significant similarity.

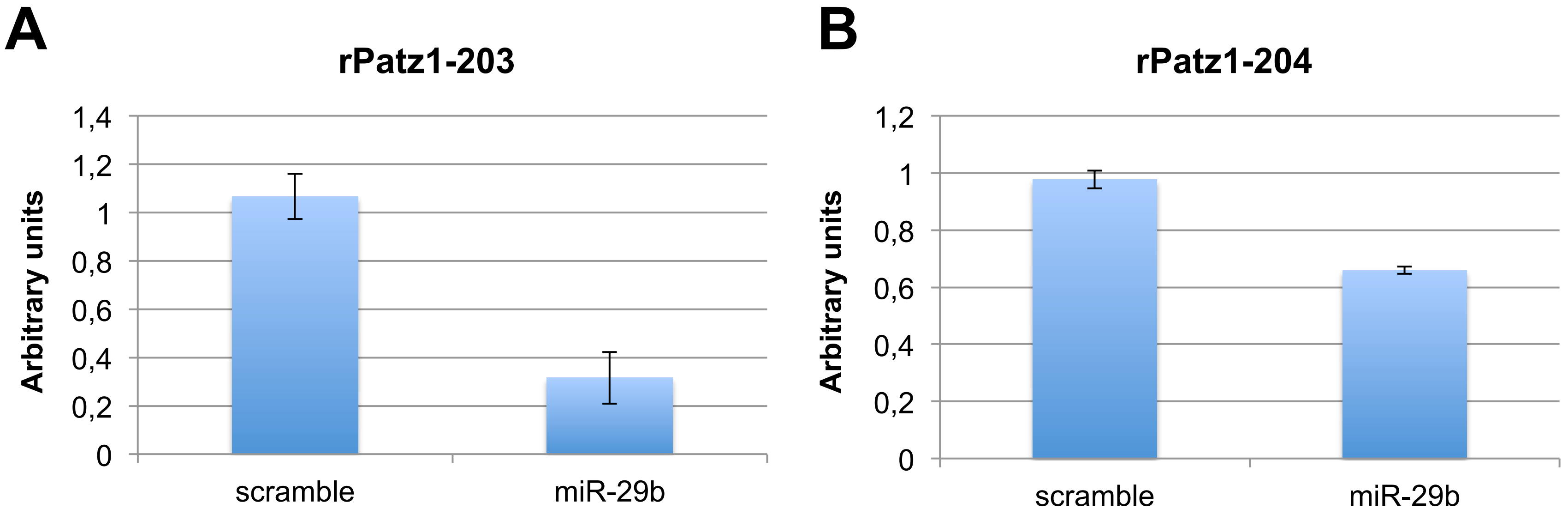

Supplementary Figure S3. miR29b effect on PATZ1 variants expressed in FRTL5 cells. qRT-PCRs using exon-specific primers able to discriminate variants rPatz1-203 (A) and rPatz1-204 (B) in FRTL5 transfected with 100 nM synthetic miR-29b precursor or scramble oligonucleotide. The mean values $\pm$ SD of a representative experiment performed in duplicate is reported.

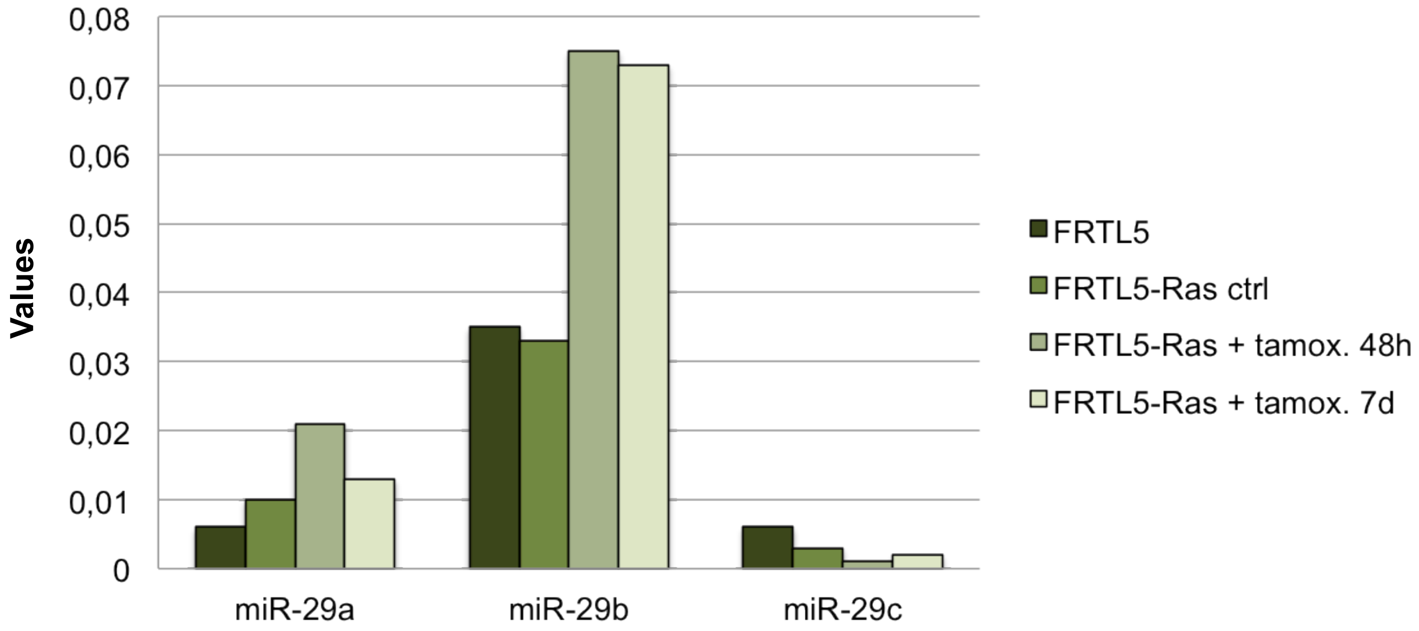

Supplementary Figure S4. miR-29 isoforms expression in FRTL5 cells and FRTL5-Ras cells as extracted by the www.microRNA.org website. In an inducible cell system in which a chimeric form of Ras oncoprotein, ER-Ras, can be activated by tamoxifen (26), microRNA profile was evaluated upon 2 and 7 days of tamoxifen treatment (28).
